# Supplementary figures and images for: A scalable culturing system for the marine annelid Platynereis dumerilii
Source: PLoS One. 2019 Dec 5;14(12):e0226156. doi: 10.1371/journal.pone.0226156 (PMC6894799; doi:10.1371/journal.pone.0226156)

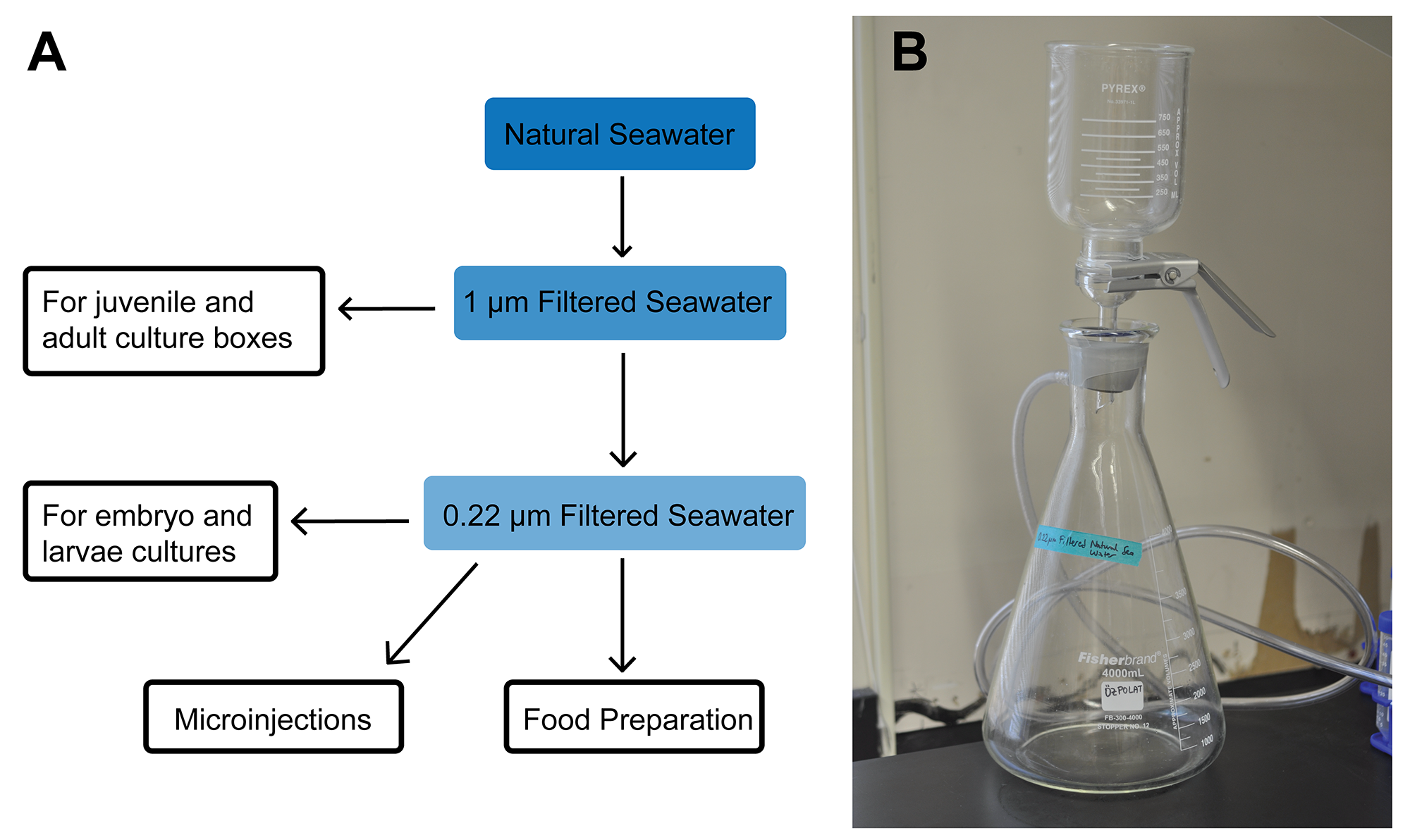

Supplement: S1 Fig — A) Schematic of seawater filtering process and what each level of filtration is used for. B) The 0.22 μm filtration set up. This system is more economical than plastic filter bottles. See supplementary spreadsheet for catalog numbers and ordering information. (TIF) [file pone.0226156.s001.tif]

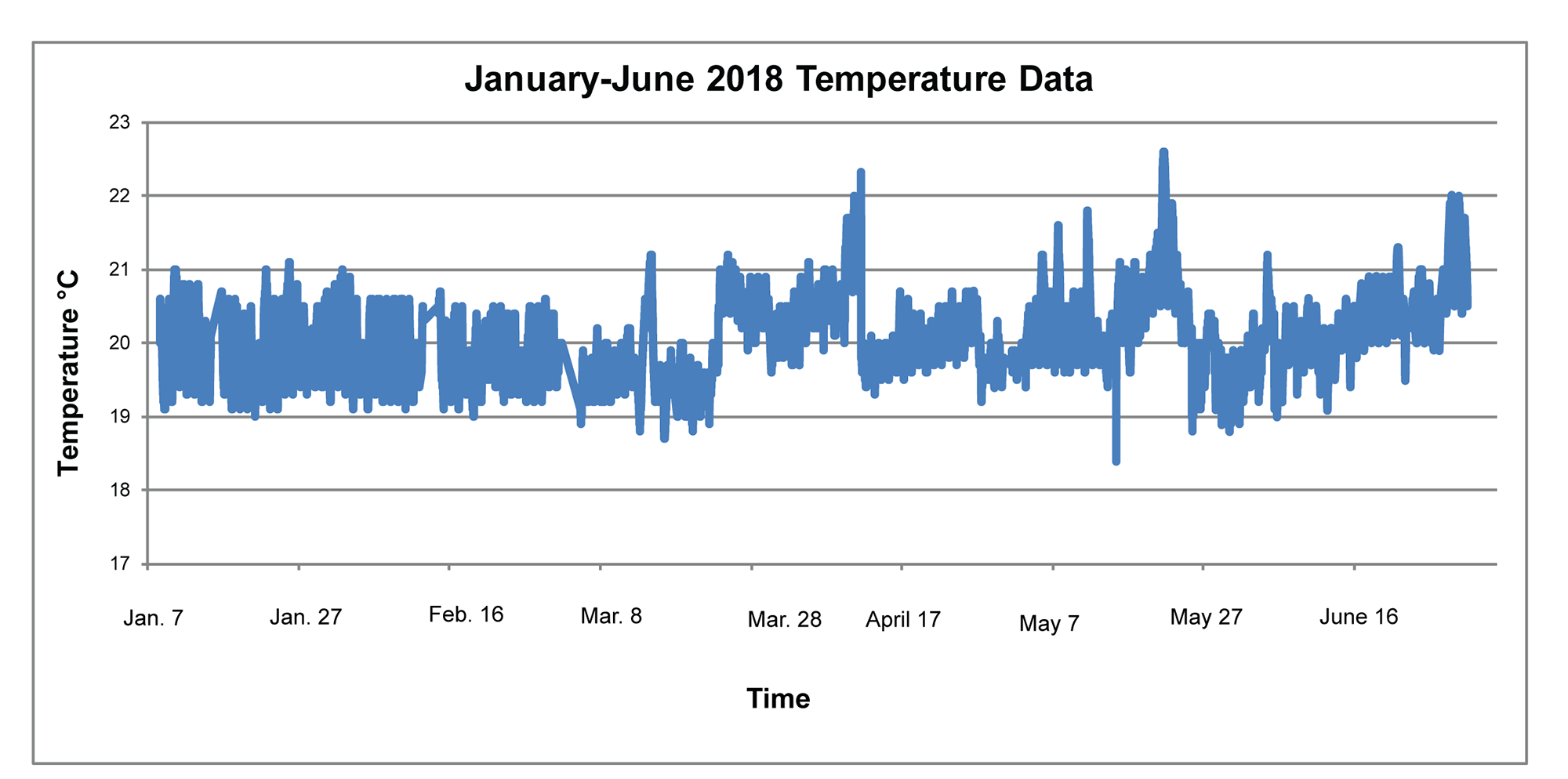

Supplement: S2 Fig — Cultures were kept at 20°C with some variability in the spring and summer months due to fluctuations in the temperature of the building. A portable air conditioning unit was used to help better regulate temperature during the summer months after the peaking of temperature above 22°C in April and May. (TIF) [file pone.0226156.s002.tif]

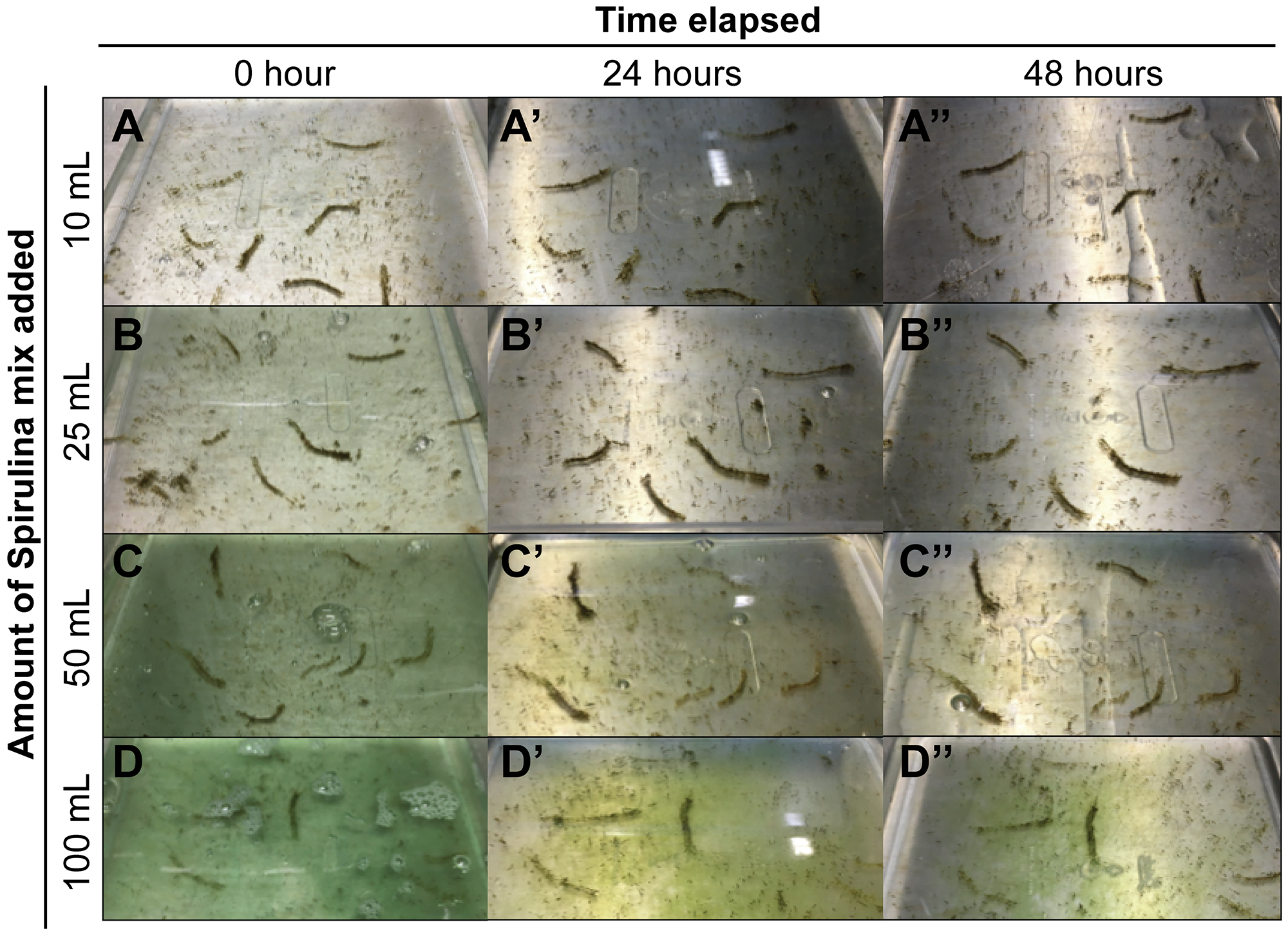

Supplement: S3 Fig — To test which concentrations of spirulina cocktail can be fed safely to a box of 10 animals without rotting and spoiling the water, four boxes of 10 animals each were fed the following amounts of spirulina cocktail: 10, 25, 50 and 100 mL. Images were taken directly after feeding, 24 hours later and 48 hours later. (TIF) [file pone.0226156.s003.tif]

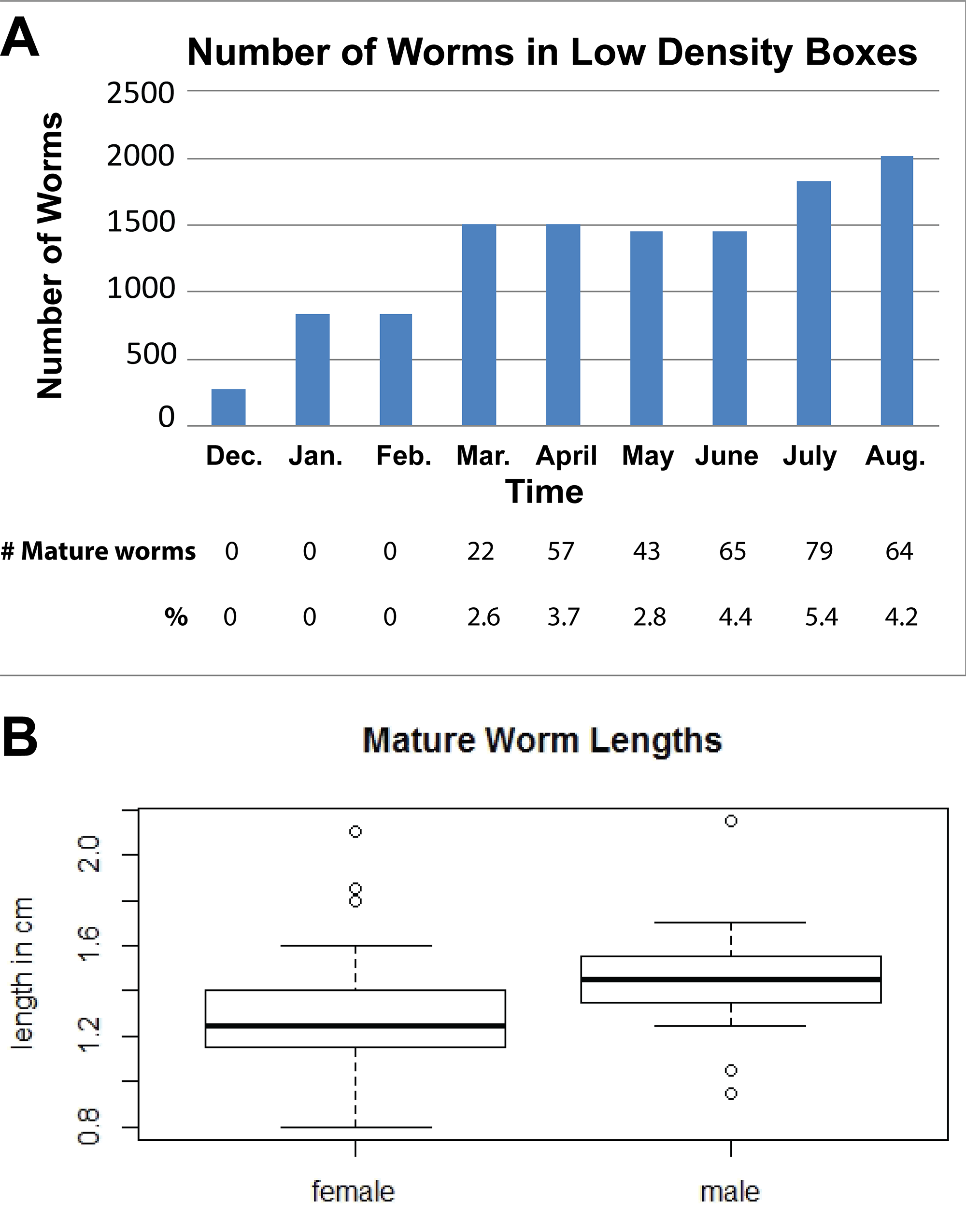

Supplement: S4 Fig — A) The graph shows the number of worms living in low density cultures (30 worms/small Sterilite box) (for the period December 2017-August 2018). The number of mature animals found each cycle increased (Fig 9D) as more low density cultures were established. These numbers (along with mature animal numbers in Fig 9) can be used as a guide to scale up or down low density culture boxes, for obtaining mature animal numbers desired. B) Mature worm size box plots from 33 females and 22 males. Worms were anesthetized in a 1:1 MgCl2 –NFSW solution for approximately 5 minutes, or until swimming stopped. They were then measured under a dissecting microscope to the nearest hundredth of a centimeter. (TIF) [file pone.0226156.s004.tif]
