# Supplementary material for: A scalable culturing system for the marine annelid Platynereis dumerilii
Source: PLoS One. 2019 Dec 5;14(12):e0226156. doi: 10.1371/journal.pone.0226156 (PMC6894799; doi:10.1371/journal.pone.0226156)
Supplement: Attachment — Submitted filename: Response to Reviewers - Kuehn et al 2019.docx [file pone.0226156.s007.docx]

**RESPONSE TO REVIEWER COMMENTS**

***We are grateful to both reviewers and the editor for their careful reviews and many constructive comments. Below we address each comment and outline the changes we have made. We thank the reviewers for their help in improving our manuscript. Each of our comments is in bold and preceded by the word "RESPONSE:"***

***All the Line numbers we refer to are for the manuscript file that has the changes tracked.***

**Editor Comments**

1) Please ensure that your manuscript meets PLOS ONE's style requirements, including those for file naming. The PLOS ONE style templates can be found at

http://www.journals.plos.org/plosone/s/file?id=wjVg/PLOSOne_formatting_sample_main_body.pdf and http://www.journals.plos.org/plosone/s/file?id=ba62/PLOSOne_formatting_sample_title_authors_affiliations.pdf

***RESPONSE: We made the necessary formatting changes across the document.***

2) We note that Figure(s) [7] in your submission contain copyrighted images. All PLOS content is published under the Creative Commons Attribution License (CC BY 4.0), which means that the manuscript, images, and Supporting Information files will be freely available online, and any third party is permitted to access, download, copy, distribute, and use these materials in any way, even commercially, with proper attribution. For more information, see our copyright guidelines: http://journals.plos.org/plosone/s/licenses-and-copyright.

***RESPONSE: We removed the panels B and C from this figure as it was not necessary to have the photos of the food packaging. We have detailed ordering information for these supplies in the manuscript and in the supplementary file 1.***

**Additional comments from editor:**
Line 194-195: What kind of algal culture medium was used? It would be useful to include the recipe or citation of the medium.

***RESPONSE: We have included details and cited a reference that will be helpful. Lines: 210-211***

Line 198: Need to provide a better specification about the desired density of the algal cultures, for example, cell numbers/ml.
 ***RESPONSE: We have provided cell numbers/mL counts for the cultures. Lines: 217-218***

Line 203: How long can this algae stocks last at this refrigerated condition?
 ***RESPONSE: We have added details into the manuscript. Line: 219***

Line 209-213: How often do you conduct this check-up?
 ***RESPONSE: We have added details. Lines: 226-227***

Figure 9 and its legend text, line 704- 714: I had a hard time understanding this figure; I cannot find the “black and white bars” from D-G. In addition, would it be possible to further calculate the percentage of total mature worms found in each month?
 ***RESPONSE: “Black and white bars” were removed from the legend (they belonged to a former version of the figure and were accidentally left in the legend). We have edited the Supplementary Figure 4 and added the number of mature worms and their percentage per month into this figure. Lines: 789-797***

Line 723-724: It would be helpful to indicate the color (cyan and magenta) for respective mRNA injection results. Confocal “microscope”.
 ***RESPONSE: We made the changes. Line: 633***

**Reviewer #1: Summary**

The manuscript “A scalable culturing system for the marine annelid Platynereis dumerilii’ by Kuehn et al. describes in detail the set up of small culturing units for this versatile annelid system. This enables any research group with access to natural seawater to rear these animals through the entire life cycle, and to control critical parameters like day/night cycles, moon cycles, light intensities, and test various feeding regiments. Thus, this set up may also open up this animal to new areas of research including various physiological and nutritional studies.
The description of the culturing method is detailed, and mostly sufficient, and the critical areas are pointed out. Although the described culturing method reaches a higher level of flexibility and control for several parameters, the authors point out where more control could/should be achieved in the future. Thus, this set up also offers a good entry point for critical improvements that can not be tackled in current larger, less flexible systems. I recommend potential publication of the manuscript after a few concerns have been addressed.

1. One parameter that is not sufficiently defined is the seawater described in the Methods as ‘full strength natural sea water’ (lane 140). Some of the experiments were performed in a laboratory in Vienna with apparently somewhat different ‘natural sea water of lower salinity’ which the authors acknowledge in lane 501, and it is important to include better definitions for the seawater here.

***RESPONSE: We have added details on the sea water salinity at both locations the experiments were carried out. Lines: 139-141***

2. The authors also mention the important observation that the jelly production by fertilized eggs at the MBL culture to be much longer compared to eggs produced by the ‘European’ cultures (lane 502). As this narrows the window for injections of zygotes significantly, it should be discussed that this might be a critical issue if the main purpose of an investigation of this annelid includes injection of zygotes. It would be important to include data that documents the extension of jelly production which seems quite dramatic (from 1h to 1.5h).

***RESPONSE: We removed the comments on the jelly production timing from the text for the following reason: When we recently tested jelly production in embryos at 1 hpf, embryos did not produce jelly beyond 1 hpf. Looks like we do not have this problem anymore, and we think this is a result of the natural fluctuations of salinity and pH in our natural sea water. We have not noticed this change before because for our experiments we inject 2-cell or 4-cell stage embryos, therefore, we typically dejelly our embryos at 1,5 hpf.***

3. ‘unknown gender worms’ (lane 224 and Figure 9): Not sure why the authors created this category as these unknown worms will mature into females and males a little later, and could be added to these gender categories accordingly? Seems unnecessary or requires better explanation.

***RESPONSE: This was necessary because of the way we collect mature worms. We place all the mature females collected into one large box, and all the mature males (and unknown genders) into another box. Lab members use the males and females from these boxes as needed for their experiments. Because we do not keep track of individual worms in individual containers, it is hard to confidently determine which sub-mature worm turned out female and which one turned out male, especially when worms are being removed from the boxes as lab members use them. We edited the text to clarify. We have also changed “gender” with “sex”, which is biologically correct term in this context. Lines: 240-254***

4. Figure 4: Black out curtain construction: As light contamination is a critical issue, I noticed that there are no Velcro strips along the bottom of the curtain (Fig 4A). Are these curtains so fitting that no light can penetrate from the bottom? How reliable is the fitting of the curtains under daily operations?

***RESPONSE: Our light sensor data shows that in the bottom shelf we do not get light contamination because the sensor reads at night show the same reading as the other shelves. If there were light contamination, we would see a higher read at nights that need to be without moon (completely dark). Therefore, we are confident that the fitting is reliable. We added comments on this in the text lines 344-347 and Figure 4 legend lines 357-361.***

5. Figure S4: Maybe this supplemental figure would be more useful by adding also the number of maturing worms or successful fertilizations each month to the figure?
 ***RESPONSE: We edited this figure and added the number and percentage of mature worms per month. Thank you for the great suggestion.***

Lane 118: .. that are affected
Lane 119: rephrase sentence
 ***RESPONSE: Thank you! Made the changes.***

**Reviewer #2:** **General comments:** This is an all-round very detailed and in-depth resource for the culturing of the important model organism Platynereis dumerilii. The paper provides a novel design for small-scale cultures that can be varied in terms of environmental conditions etc.
Overall, this manuscript provides a lot of data to support the successful establishment of a small-scale culturing system which is able to produce mature individuals in good numbers. I believe it to be an excellent starting place for those looking to establish cultures of their own at whatever scale and it provides important data to support future efforts for standardisation of culturing techniques across laboratories working with this species.

**Minor comments:**

1) I feel that the standardisation of feeding method by using the spirulina diet shows promise however the authors should make it clear that this method is not yet fully optimised. The results presented do not provide enough information to give a comparison between this and traditional feeding methods due to feed volumes which may or may not contribute to inferior growth rates and the different sites of the experiments. The animals on the spirulina diet were not fed ad libitum.
 ***RESPONSE: We believe using spirulina diet perfectly replaced the traditional methods at our lab at the MBL, as we get healthy mature animals that we have successfully used for experiments. In that sense, we think the use of this particular food source is optimized, it is more accurate to weigh, and the recipes provide a standardized reference point for other labs. However, there are many other parameters in addition to food that make each lab’s culturing conditions slightly different (for example, the degree of natural sea water used, and the degree of algae naturally present in the sea water can change how much food is available to the animals). Because of these differences, labs may have to increase or decrease the amount of spirulina provided to the animals. The key point here is, however, they will be able to report the exact amounts of spirulina they are feeding the animals with. For experiments in which nutrient availability should be precisely controlled, in addition to using spirulina, researchers should control for other conditions that may change the nutrient availability. While this is an important point, we believe it does not mean that spirulina feeding method is not optimized.***

2) It would be useful if the authors mentioned the potential impact of several ‘unseen’ water quality parameters such as pH, nitrites, nitrates, ammonia; all of which would impact the long term health and prosperity of the culture. These parameters will potentially all be influenced by the feeding regime.
***RESPONSE: We have added comments on this in the Future Directions section. Lines: 649-652***

3) Do the authors also have data about the size of the worms at maturity? It would be useful to add this to the paper, if such data exist.
***RESPONSE: We added this information into Supplementary Figure 4 as a box plot. We also added a sentence about the mature worm size into the text. Lines: 610-612***

4) 100 "transgenesis and genetic tools" - it would be good to mention that homozygous knockout lines have been established, first with ZFN, then TALEN, and more recently with CRISPR, and cite the relevant papers
5) 101 "behavioral tracking [8,16]" - under behavioral tracking, it would be good to mention work also on the larval stages

6) 101 "and live imaging [5] - please cite also work on neuronal activity imaging
***RESPONSE: We have added the relevant papers. Lines: 94-96***

7) 118 “This is particularly important for studying biological processes are affected dramatically by nutrition” incomplete sentence
***RESPONSE: Fixed, thanks!***

8) 177-185: What is the size / what are the dimensions of a large box? And what volume of water is added?
***RESPONSE: This information is in the Supplementary File 1. To make it more accessible, we added it into the methods section in the manuscript. Line: 202***

9) 182-184: What bubbling rate was used as this could impact water quality or organism behaviour. If this was not measured directly, was it gentle / vigorous etc?
***RESPONSE: We added details to clarify. Line: 194-196***

10) 223-231: Please define ‘mature worms’ here
***RESPONSE: We changed the text to have better clarity and definitions. Lines: 240-250.***

11) 288-290: You mention that you ‘stack’ boxes on a shelf – do you have any information as to whether this impacts culture conditions, for example light levels for the top box in the stack vs the bottom?
***RESPONSE: We measure the light levels in stacked up boxes and found that light penetrates enough to the bottom boxes to help with worm maturation. We added the following into the manuscript:*** *We placed the sensor in an empty box and placed a culture box on top to test if moonlight penetrated stacked cultures. We found that moonlight levels were lower (typically around 5 lux), but slightly above our “zero” value (3-4 lux). We did not systematically keep track of how, or if, the reduced moonlight affected worm maturation of stacked cultures, however we did not notice any differences in mature worm production by these boxes.* ***Line: 431-436***

12) 325: Please reference who the ‘others’ are that you followed a standardised protocol from.
***RESPONSE: Reference was added. Line: 107***

13) 423-427: Do you have any additional information or opinions on why the algal growth may have been different when comparing the two locations? For example, different sources of seawater / different filtration etc?
***RESPONSE: We added the following to the text:*** *The source of this algal growth is likely due to differences in seawater sources. Also, juvenile worms in Vienna cultures are kept in 1:1 artificial and natural sea water. This potentially dilutes the amount of algae introduced into each culture box.* ***Lines: 529-532***

14) 455-456: Do you have any data or quantification to reinforce the ‘appearance’ of a larger size of larvae?
***RESPONSE: We have not carried out measurements. We edited the sentence and removed this comment as we cannot provide quantification. Lines: 559-560***

15) 466-467: In how many boxes / what culture size are this number of mature worms being produced?
***RESPONSE: We have edited the Supplementary Figure 4 and added the number of mature worms and their percentage per month into this figure. Considering we use 30 worms per small culture box, the approximate number of boxes can be calculated with all the information now provided in this figure.***

16) 470-473: What lighting regimen were the initial boxes on if they were not on the one you have detailed?
***RESPONSE: We have added information on the lighting regimen for the initial boxes. Lines: 577-578***

17) 474-475: For these 22 mature worms, what timescale is this over? A day? A cycle?
***RESPONSE: Detail added to the text. Line: 580-581***

18) 501-503: The authors mention differences in salinity but what salinities are these and how large is the difference between the two?
***RESPONSE: We added salinity information for both MBL and MFPL into the methods. Lines: 138-141***

19) 511-514: I feel this needs to be discussed in a little more detail as the authors haven’t mentioned the inability to culture in artificial sea water before this point. Is there any data / information / references which compare natural and artificial sea water or discuss not being able to culture Platynereis in artificial sea water?

***RESPONSE: We have added more discussion into the Future Directions section on this topic. Lines: 642-646***
